# Redox Dysregulation in Aging and COPD: Role of NOX Enzymes and Implications for Antioxidant Strategies

**DOI:** 10.3390/antiox10111799

**Published:** 2021-11-11

**Authors:** Caspar Schiffers, Niki L. Reynaert, Emiel F. M. Wouters, Albert van der Vliet

**Affiliations:** 1 Department of Pathology and Laboratory Medicine, University of Vermont, Burlington, VT 05405, USA; caspar.schiffers@lunghealth.lbg.ac.at (C.S.); woutersemiel@gmail.com (E.F.M.W.); 2 Ludwig Boltzmann Institute for Lung Health, 1140 Vienna, Austria; 3 Department of Respiratory Medicine, NUTRIM School of Nutrition and Translational Research in Metabolism, Maastricht University Medical Center, 6211 LK Maastricht, The Netherlands; n.reynaert@maastrichtuniversity.nl

**Keywords:** ageing, COPD, oxidative stress, redox signalling, NADPH oxidase, DUOX1, antioxidants

## Abstract

With a rapidly growing elderly human population, the incidence of age-related lung diseases such as chronic obstructive pulmonary disease (COPD) continues to rise. It is widely believed that reactive oxygen species (ROS) play an important role in ageing and in age-related disease, and approaches of antioxidant supplementation have been touted as useful strategies to mitigate age-related disease progression, although success of such strategies has been very limited to date. Involvement of ROS in ageing is largely attributed to mitochondrial dysfunction and impaired adaptive antioxidant responses. NADPH oxidase (NOX) enzymes represent an important enzyme family that generates ROS in a regulated fashion for purposes of oxidative host defense and redox-based signalling, however, the associations of NOX enzymes with lung ageing or age-related lung disease have to date only been minimally addressed. The present review will focus on our current understanding of the impact of ageing on NOX biology and its consequences for age-related lung disease, particularly COPD, and will also discuss the implications of altered NOX biology for current and future antioxidant-based strategies aimed at treating these diseases.

## 1. Introduction

Human life expectancy has nearly doubled globally during the past century, and the global human population over the age of 65 is expected to represent ~20% of the world’s population by 2050 [1]. Ageing is characterized by gradual and irreversible functional deterioration of all vital organs after the reproductive phase of life is complete [2], and is a major risk factor for death from all adult chronic diseases. Therefore, the rapid increase in the ageing population together with declining fertility rates will create an ever-increasing societal burden and health care challenge over the next decades, and demands for increased understanding of the molecular mechanisms underlying ageing and age-related disease, enabling advanced health care for our elderly, will become increasingly urgent.

On a cellular and molecular level, Lopez-Otín and colleagues have defined nine hallmarks of ageing, which include genomic instability, telomere attrition, epigenetic alterations, loss of proteostasis, deregulated nutrient sensing, mitochondrial dysfunction, cellular senescence, stem cell exhaustion, and altered intercellular communication [3]. Dysregulation of the extracellular matrix due to aging is an additional crucial modifier of cell-autonomous changes and functions [4]. The origin of these various hallmarks is undoubtedly complex, and is likely to involve a combination of underlying processes that may be cell- and organ-specific, and the individual contribution of each hallmark to individual ageing-related non-communicable chronic diseases may vary.

One well-recognized aspect of ageing is the enhanced production of reactive oxygen species (ROS), which are generated during cellular metabolism of molecular O_2_ and lead to accumulation of biomolecular oxidative damage [5,6,7]. This, combined with the lifelong exposure to ionizing radiation or environmental oxidizing pollutants, has led Denham Harman to propose the free radical theory (FRT) of ageing [8]. This theory was later refined to the mitochondrial free radical theory of ageing, based on the fact that mitochondria are the primary source of ROS, and in line with mitochondrial dysfunction as one of the main hallmarks of ageing [3,9]. Evolutionary evidence does not always support the FRT of ageing, however, and the recognition of physiological functions of ROS in e.g., host defense and other aspects of cell biology through redox-based signalling has further complicated the FRT of ageing. Indeed, the recently discovered family of NADPH oxidase (NOX) enzymes are critical in these physiological roles, and the association(s) between NOX function and redox-based signalling and ageing are only beginning to be appreciated (e.g., [10,11]).

The lung is the organ with the largest surface area that faces the external environment, estimated to be as large as half a tennis court [12], and is therefore exceptionally vulnerable to the life-long exposure to environmental pathogens and common (oxidizing) airborne pollutants. Indeed, ageing is associated with a progressive decline in lung function and with increased susceptibility to the development of chronic age-related pulmonary diseases, such as chronic obstructive pulmonary disease (COPD) and idiopathic pulmonary fibrosis (IPF), all rapidly increasing in incidence with advancing age [13,14]. Likewise, oxidative stress (due elevated levels of ROS and/or impaired antioxidant defenses) is often viewed as a common feature of, and contributor to these diseases [15], and has encouraged the proposed use of antioxidant-based strategies in potential treatment of these diseases. In the present review, we will summarize the general physiological features of lung ageing, and the pathogenesis of one important age-related lung disease, COPD, which currently represents the third leading cause of death in the Westernized world [16]. We will then revisit the FRT of ageing with a specific emphasis on the relationship with NOX family enzymes, and the relevance for oxidative stress in age-related lung diseases such as COPD. Lastly, we will present an overview the current status of antioxidant-based strategies to mitigate age-related lung disease, and discuss potential implications for recently observed alterations of NOX functions during ageing.

## 2. The Ageing Lung

Throughout human lifespan, various age-associated structural and functional changes occur within the respiratory system, termed the lung function trajectories. Lung growth occurs from birth until adulthood and is characterized by increases in lung volume, an increase in the number of alveoli, and increased capillary networks [17]. During the plateau phase (adolescence ~25 years of age to 30–40 years) these numbers remain stable [18], after which [19], lung function starts to gradually decline with increasing age, which may be variable in every individual based on genetics and different exposure histories to e.g., cigarette smoke or other environmental challenges. It is important to recognize that individual lung growth may vary as well, and that abnormal lung growth early in life may also affect later phases of lung function trajectories [20,21].

The decline phase has various consequences for functional capacity in absence of underlying pathology and affects every individual, with early limitations only observed during exercise and later on during broader settings. Accordingly, with advancing age, the respiratory tract undergoes both structural and physiological changes, such as loss of lung regenerative capacity and pulmonary remodelling, which are associated with a progressive decrease in lung function [22]. Characteristic of the ageing lung is a decrease in lung elasticity and concomitant increase in alveolar size. This loss in lung elasticity and airway enlargement results in increased functional residual capacity (FRC) and end-expiratory lung volume (EELV). Additionally, the ratio between the forced expiratory volume in one second and forced vital capacity (FEV1/FVC), often used to diagnose chronic obstructive lung diseases and defined as the amount of air that can be forcibly exhaled following one’s maximal inhalation, decreases with age due to loss of lung elasticity and airspace enlargement, and also because of loss of respiratory muscle mass [18]. Also termed the ‘senile lung’, these age-related structural lung changes are mainly attributed to an increase in the size of the alveolar space and are not considered pathological because they occur in the absence of significant inflammation or alveolar wall destruction.

The respiratory epithelium also undergoes age-related structural and functional alterations. This is evidenced by reduced mucociliary clearance in the upper and lower airways, reductions in relative numbers of basal cells, stem cell senescence, alterations in airway and alveolar epithelial differentiation, increased transcriptional noise due to declined epigenetic regulation, and impaired epithelial responses to injurious triggers [23,24,25,26,27]. These changes collectively impact on appropriate epithelial responses to environmental pathogens, although the precise impact of ageing on these processes is still incompletely understood. For example, acute airway inflammatory responses to microbial stimuli are somewhat attenuated with age and appear prolonged [28]. In apparent contrast, ageing dramatically impacts on innate airway epithelial responses to non-microbial injurious triggers, including airborne allergens such as house dust mite, as shown by markedly reduced acute production of epithelial alarmins and subsequent type 2 immune responses that are critical for regenerative processes in response to injury [29]. It is plausible that such impaired epithelial regenerative responses may also contribute to senile emphysema.

In addition to negatively impacting lung structure and physiology, ageing is known to lead to a gradual dysregulation of the immune system, which is characterized by an impaired ability of various immune cells to respond to pathogens, and by age-related low-grade inflammation due to immunosenescence (known as inflammageing) [23,24,30]. Replicative and/or stress-induced cellular senescence of immune cells results in compromised and inappropriate cellular function and cell responses of e.g., innate and humoral immunity [31]. This is largely responsible for the increased susceptibility of elderly subjects to infection with influenza virus or with SARS-CoV-2 [32,33,34]. Replicative senescence of resident cells induces the senescence-associated secretory phenotype (SASP), which is characterized by resident senescent cells secreting pro-inflammatory factors that can alter the cellular microenvironment and shift neighbouring healthy proliferating cells into a more senescent- and pro-inflammatory state. In addition to damage-associated molecular patterns (DAMPs), the SASP contributes to inflammageing/sterile inflammation observed during lung ageing [35], which is characterized by pro-inflammatory cytokine release and chronic low-grade inflammation in the absence of an immunological threat [36]. While associated with ageing, the SASP has likely evolved as a mechanism to maintain homeostasis through senescent cell clearance, progenitor cell repopulation, and wound healing and tissue repair [31,37,38], and has also been shown to counter early-life tumorigenesis [39].

Because the respiratory system represents a critical interface with the external environment and is susceptible to injury from inhaled environmental pathogens and pollutants, it is equipped with various defense mechanisms (antioxidant defenses, antimicrobial defenses, mucus and mucociliary clearance mechanisms, and local sentinel immune cells). Age-related decline in these mechanisms likely contributes to biochemical and physiological changes in the lung, and may contribute to the development of age-related chronic lung disease(s) [40,41,42]. Indeed, while the senile lung is characterized by airspace enlargement in the absence of overt inflammation and tissue remodelling, such compromised and inappropriate responses to exogenous hazards in the ageing lung likely contribute to chronic inflammation and alveolar wall destruction that contribute to the development of e.g., emphysema [43,44], and also render the aged lung more susceptible to acute injury or infections that contribute to exacerbations, and may in turn further aggravate lung ageing. Hence, the molecular mechanisms underlying chronic lung diseases such as COPD are also dictated by alterations that occur as a result of normal aging. Recent efforts using single-cell transcriptomics and proteomics to develop an atlas of normal aging, such as the Tabula Muris Senis database [45], have dramatically increased our insights into (lung) cell-type specific effects of ageing [46], and present highly useful resources to assess the contributions of ageing to chronic age-related diseases, including those of the lung.

## 3. Oxidative Stress in Ageing: Revisiting the Free Radical Theory (FRT) of Ageing

In the 1950’s, Denham Harman recognized that many manifestations of ageing resemble effects of chronic ionizing irradiation, and since both involve the formation of damaging ROS such as hydroxyl radicals (OH^●^), he first proposed the FRT of ageing, which essentially poses that accumulation of oxidative biomolecular damage with advancing age contributes to functional decline. Since its initial proposal, the FRT of ageing gained ample experimental support, but various lines of experimental evidence are also inconsistent with this proximate theory of ageing [47], as is summarized in Table 1.

For example, strong correlations have been observed between chronological age and levels of ROS generation or accumulation of (irreversible) markers of oxidative stress, such as increased rates of (mitochondrial) ROS production, gradual decreases in glutathione/glutathione disulfide (GSH/GSSG) or cysteine/cysteine redox states [48,49,50,51], and age-related accumulation of irreversible oxidation products in proteins or DNA. Indeed, irreversible oxidative damage to nuclear and mitochondrial DNA is significantly increased in all major tissues in aged organisms, including mice and rats [55] as well as humans [56,57], and also in lung tissues [58,59]. Also, substantially higher levels of DNA damage and lipid peroxidation products (e.g., lipid peroxides and malondialdehyde (MDA)) have been observed in the lungs of aged organisms when compared with young counterparts [58,59]. Age-related aggregation of mitochondrial damage can cause accumulated mutations in mtDNA, which is particularly susceptible to ROS-induced damage [64], perpetuating enhanced ROS generation in mitochondria, which may be relevant for chronic diseases associated with ageing. Gender divergence in mice with respect to ageing was also found to be associated with indices of oxidative stress with females being shorter-lived and having greater increases with age [66]. Finally, biological ageing is also associated with a gradual decrease in tissue expression of antioxidant and oxidoreductase enzymes [65], which would enhance susceptibility to oxidative stress with advancing age. Such age-related decline in antioxidant mechanisms is largely associated with decreased adaptive response to oxidative stress, for example by diminished activation of the transcription factor nuclear factor erythroid 2–related factor 2, or Nrf2, a master regulator of antioxidants, metabolic enzymes, and anti-inflammatory genes [67]. Indeed, while Nrf2 expression was shown to be increased in bronchial epithelial cells with increasing age, potentially as a result of increased steady-state levels of oxidation, the inducibility of Nrf2-mediated antioxidant responses was compromised in the context of ageing [73,74,75]. The increased expression of Nrf2 suppressors such as Bach1 and c-Myc may contribute to the impaired inducibility of the Nrf2-regulated antioxidant genes.

In spite of these various lines of evidence, other observations are inconsistent with the FRT of ageing and illustrate that this theory is too simplistic. For example, there is a lack of correlation between levels of ROS and longevity across various animal species. An intriguing example of this is the naked mole rat, a rodent with a lifespan well beyond prediction for its size, which maintains normal activity, body composition, and reproductive and physiological functions with no obvious age-related increases in morbidity or mortality rate for over the majority of their lives [52]. Yet, naked mole rats display relatively high levels of oxidative stress, elevated levels of oxidative damage, and less robust repair mechanisms than their shorter-lived rodent counterparts [53,54]. Also, some mutant organisms associated with mitochondrial dysfunction (such as *C. elegans* NADH dehydrogenase ubiquinone flavoprotein 1 (nuo-6) mutant or mitochondrial 5-demethoxyubiquinone hydroxylase (Mclk1) mutant mice) have a longer lifespan compared to their wild-type counterparts, which may in fact be due to increased levels of mtROS production [60,61]. Indeed, relatively low levels of mitochondria-derived ROS, especially in early life, are essential for improving systemic defense mechanisms by inducing adaptive responses and thereby promote healthspan, a concept known as mitochondrial hormesis (mitohormesis) [62,63].

Also arguing against the FRT of ageing are several studies with antioxidant supplementation strategies to reduce oxidative stress, which have either failed to enhance longevity [76] or even reduced it [69]. Similarly, genetic manipulation of 18 different genes involved in antioxidant defense in mouse models showed no effect on lifespan, except for the Sod1 gene, which encodes mitochondrial manganese superoxide dismutase (MnSOD) and is essential for maintaining mitochondrial function [70,71]. As another example, constitutive overexpression of the antioxidant protein thioredoxin (Trx) 1 in ageing mice showed no life-extending effects in the later part of life (>22–25 months) and was in fact associated with enhanced tumor development at that age, due to suppression of the ASK1 pathway [77]. Overexpression of both cytosolic Trx1 as well as the mitochondrial isoform Trx2 was in fact found to significantly shorten lifespan compared to wildtype mice, and increased incidence and severity of lymphoma [78], consistent with the mitohormesis concept and suggesting that antioxidant overexpression or supplementation can also adversely affect age-related diseases, especially cancer. A recent systematic analysis of protein cysteine oxidation networks in various mouse tissues indicated that redox-regulated sites and networks vary between different tissues and are fundamentally remodelled in the context of ageing, with some cysteine oxidations actually decreasing with age, contrasting the prevailing thought that ROS-mediated protein oxidation indiscriminately increases with age [10]. Given the highly variable nature of oxidative post-translational cysteine modifications and their often-unknown functional consequences, it is difficult to predict how such specific age-related changes impact on lung ageing or age-related disease. The recently emerging gaseous signalling molecule hydrogen sulphide (H_2_S) is believed to positively impact lifespan and ageing, which may be related to its ability to mediate a particular oxidative protein cysteine modification, S-sulfhydation (or persulfidation), which was recently found to decline with aging [72]. These observations indicate that some oxidative modifications may actually be associated with longevity, and clearly illustrate the severe limitations of the simplistic concept of the FRT of aging. The pleiotropic nature of biological ROS sources, including dedicated enzymes such as NOX, and our relatively limited understanding of the effect of ageing on these enzyme systems (as will be discussed further below), further complicates this issue.

Alternative theories of ageing have been proposed that primarily focus on the question why we age, more so than the proximate FRT of aging that may explain how we age. These include the disposable soma theory [79], which suggests that ageing is a trade-off in the allocation of limited energy resources between maintenance and restoration of tissue homeostasis, and other traits needed for survival (e.g., reproduction); and the related antagonistic pleiotropy (AP) theory of ageing [80], originally proposed in 1957 by Williams, which poses that genes that confer a reproductive advantage early in life may have harmful effects later in life [81]. The AP theory is potentially applicable to metabolic genes that impact on mitochondrial ROS production, and would be consistent with the mitohormesis concept of promoting health span in early life while also contributing to ageing due to mitochondrial dysfunction. The AP theory has also been suggested to apply to NOX enzymes, as they play critical roles in e.g., host defense or developmental aspects which would be critical in early life, but may also be detrimental later in life if they are inappropriately activated during ageing or age-related disease [82]. However, as will be discussed in the next sections, associations of NOX enzymes with ageing or with age-related lung disease are not always uniform and do not necessarily conform to these ultimate/evolutionary ageing theories.

## 4. COPD a Disease of Accelerated Ageing?

COPD is a chronic irreversible disease of the lungs characterized by airflow limitation due to destruction of the lung parenchyma (emphysema) and/or remodelling of the small airways, and is currently the third leading cause of death in the Westernized world [16]. The greatest risk factor for COPD is smoking, but not all smokers develop COPD and the reasons for disease susceptibility in these individuals remains poorly understood [83]. In COPD, the alveolar architecture has been destroyed resulting in emphysema [84,85] and subsequent dyspnea (shortness of breath). Small airway disease and emphysema development are mechanistically related, since small airway inflammation may propagate to the alveolar septa, in turn destroying bronchiolar-alveolar attachments, and eventually proceed into lung parenchymal destruction [86,87,88]. Moreover, a loss of small airways before the onset of parenchymal destruction may explain the increased peripheral airway resistance described in COPD [85]. Another histopathological feature often observed in COPD patients is seen in the vasculature with increased thickness of the arterioles, resulting in pulmonary hypertension as an additional complication of COPD [89].

Various genetic factors have been established as risk factors for COPD, such as genetic defects in the *SERPIN1* gene resulting in alpha1-antitrypsin deficiency [90,91]. The most widely recognized cause of COPD pathogenesis is however exposure to repeated environmental insults such as tobacco smoke, which is associated with repetitive injury and persistent inflammation and imbalanced protease/anti-protease activities within the lung [92,93], thus leading to progressive lung tissue damage, abnormal tissue remodelling, and tissue fibrosis [94]. As a result, COPD is characterized primarily by thickening of (large and small) airways due to subepithelial fibrosis and mucus plugging, and a related obstruction of the small airway lumen, and with alveolar emphysema due to alveolar wall destruction and loss of alveolar surface area.

At the cellular and molecular level, COPD is characterized by various alterations of cell biology such as telomere shortening [95,96] and senescence/SASP in many cell types [97], including endothelial [98] and (alveolar and bronchial) epithelial cells [99], as well as fibroblasts [100]. Furthermore, COPD is characterized by altered/impaired innate immune function that may contribute to infection and exacerbations in this disease. These various alterations and decline in function are greatly impacted by cigarette smoking [95,99]. For example, cigarette smoking may contribute to basal cell hyperplasia as one of the initial events of altered epithelial cell biology in COPD [101]. Such alterations in the basal cell population also contribute to airway epithelial remodelling phenotypes including mucous cell hyperplasia, epithelial-mesenchymal transition (EMT), altered cell differentiation, and impaired epithelial barrier integrity [102,103].

More recent transcriptional profiling studies of airway basal cells from COPD patients and non-COPD controls revealed a marked heterogeneity indicating a continuum of basal cell status that may represent gradually evolving trajectories of basal cell phenotypes as COPD develops [104]. Transcriptional analyses also indicated that smoking can induce a distal-to-proximal repatterning of small airway epithelial cells, which was attributed to increased activation of the epidermal growth factor (EGF)/epidermal growth factor receptor (EGFR) pathways [105]. Transcriptional analysis also suggested a reprogramming of alveolar macrophages in COPD, with relatively less M1 polarization and a shift towards partial M2 polarization. These alterations appear to correlate with COPD severity [106], and to be driven by oxidative stress induced by smoking, as they lead to impaired innate macrophage activation in response to e.g., infection [107,108]. Mechanistic studies suggested a potential for acrolein, a major electrophile of CS, in such macrophage alterations, due to the reactivity of acrolein towards thiols within critical proteins involved in macrophage activation/polarization such as nuclear factor kappa-light-chain-enhancer of activated B cells (NF-κB) and c-Jun N-terminal kinase 2 (JNK2) [109]. COPD may develop through variable lung function trajectories. Indeed, while some COPD patients may display accelerated age-related lung function decline following normal lung growth, others show evidence of abnormal lung growth with normal age-related lung function decline [20,21]. Furthermore, the ageing lung is characterized by ‘senile emphysema’, which is characterized by a loss of elasticity, enlargement of alveoli as well as low-grade inflammation. However, it is not a result of destruction of the alveolar walls, which does underlie emphysema in COPD. Cellular senescence is observed during lung ageing and may suggest a predisposition to COPD development. As such, examining and understanding the underlying molecular mechanisms involved in normal lung ageing (e.g., based on available insights from public databases such as Tabula Muris Senis) may help to understand how tobacco smoke and other oxidative stressors may accelerate lung ageing and result in COPD development. Indeed, many of the known hallmarks of ageing are also thought to contribute to COPD pathogenesis, such as epigenetic alterations (e.g., due to dysregulation of histone deacetylases), loss of proteostasis (regulation of protein biogenesis, folding, trafficking and degradation), mitochondrial dysfunction, and cellular senescence [3,4]. Altered intercellular communication (e.g., adaptive immune responses), and abnormal extracellular matrix (ECM) turnover and deposition further contribute to COPD pathogenesis [4].

Another aspect worth highlighting is the heterogeneity of COPD diagnosis and the diverse descriptions of COPD in the literature. Indeed, while spirometry is recommended by Global Initiative for Chronic Obstructive Lung Disease (GOLD) to diagnose COPD [110], there is ongoing debate regarding the diagnosis and definitions of COPD [111], as large cross-sectional studies focusing on COPD often have variable definitions of COPD (diagnosis). In this light, a large COPD cohort study, COPDGene, developed an integrated approach for COPD diagnosis [112], using environmental exposure, clinical symptoms, computed tomography (CT) imaging and spirometric criteria. These data have important implications for defining COPD and recognizing phenotypes, and will also be important in future therapies that are likely to be most effective in early COPD [111].

## 5. Oxidative Stress in COPD: The Present Evidence

Over the past decades, it has been widely appreciated that COPD is strongly linked with increases in oxidative stress [92,113]. Among the first lines of evidence are studies in the 1960s, describing that individuals with alpha-1-antitrypsin deficiency developed emphysema early, based on oxidant-induced inactivation alpha-1-antitrypsin especially in smokers [114]. Indeed, chronic exposure to cigarette smoke (or e.g., combustion products of biomass fuels [115]) has been linked to marked increased levels of oxidative and carbonyl stress in the lungs [116,117] that affect many of the hallmarks of ageing (e.g., senescence, genomic instability, epigenetics), resulting in ‘accelerated’ ageing of the lungs that underlies COPD pathogenesis [118]. Oxidative stress due to cigarette smoke exposure may also contribute to telomeric DNA damage foci, thereby inducing cellular senescence and promoting the development of lung emphysema [119]. Characteristic redox imbalances and markers of oxidative stress in COPD include elevated concentrations of nitrotyrosine in lung inflammatory cells and various lipid peroxidation products (8-isoprostane, 4-hydroxy-2-nonenal) in serum and lung tissue [120], elevated levels of e.g., MDA [121], and during acute exacerbations elevated production of ROS by alveolar macrophages [122]. Moreover, exhaled breath condensates from COPD patients show increased concentrations of H_2_O_2_ and myeloperoxidase (MPO) [120]. DNA damage as well as lipid peroxidation are also elevated in smokers, highlighting the significance of cigarette smoke (CS) in oxidative damage [58] and COPD. Mitochondrial dysfunction is another major contributor to oxidative stress/redox imbalance in COPD [123], and has been observed in epithelial cells [124] and airway smooth muscle cells from COPD patients [125]. Furthermore, altered antioxidant defense systems are also observed in COPD that may further contribute to oxidative stress and/or redox imbalance [123]. For example, MnSOD is elevated in the alveolar epithelium of cigarette smokers and likely due to an increased oxidant burden in these subjects [126], whereas extracellular superoxide dismutase (EcSOD) is differentially expressed in COPD patients being elevated in sputum, but reduced in e.g., bronchioles [127]. Lastly, oxidative stress in COPD is not only observed in the lungs, but is also observed systemically, including blood plasma. Indeed, red blood cells (RBCs) are affected by oxidative stress and undergo senescence in COPD due to oxidative modifications [128]. The same group has also demonstrated that N-acetylcysteine counteracts RBCs alterations (cytoskeletal structure) in COPD [129]. Additionally, oxidative stress is known to be involved in cachexia/muscle wasting, a well-recognized systemic feature of COPD [130,131]. While these aspects are certainly relevant, the current review deliberately focused on localized effects of oxidative stress to the lungs.

Because of the strong link of COPD with cigarette smoking, and the abundant presence of (oxidizing) radicals in cigarette smoke, it is often assumed that oxidative stress in COPD is a direct result of smoking. However, the “oxidative stress” induced by CS is largely due to unsaturated aldehydes contained therein (e.g., acrolein) which are primarily responsible for reactions with cellular and extracellular thiols and thiol-containing proteins [132,133,134,135,136]. In turn, CS-derived aldehydes can also induce oxidative stress more indirectly by inducing mitochondrial dysfunction, and by diminishing antioxidant function. It is also important to point out that COPD is known to progress even after smoking cessation [137], probably related to the fact that smoke exposure can leave a long-term signature of epigenetic changes such as alterations in DNA methylation [138]. Also, oxidative stress in COPD is increased particularly during acute exacerbations, which are associated with increased inflammation and infiltration/activation of neutrophils and macrophages. As such, it is likely that the biological responses induced by initial smoking (e.g., inflammation, epigenetic alterations) are involved in redox alterations and disease progression rather than smoke-derived oxidants themselves. Also, redox alterations in COPD may in part include changes occurring as a result of “normal” ageing (e.g., mitochondrial dysfunction, changes in antioxidant defenses), which may be accelerated or enhanced as a result of smoking and inflammation during e.g., exacerbations.

Related to observations of impaired antioxidant defenses in COPD, the activity of Nrf2 has been observed to decrease in mouse models of COPD [139] as well as in COPD patients [140], thereby resulting in suppressed Nrf2-mediated antioxidant and cytoprotective gene expression. In line with this, increased Nrf2 activity through activation of canonical Wnt-β-catenin signaling in mice was found to exert protective effects on lung inflammation and elastase-induced emphysema [141]. In apparent contrast, concentrations of the antioxidant co-factor GSH in epithelial lining fluids (ELF) were reported to be elevated in chronic smokers compared to nonsmokers, implying that smoking itself induces GSH-dependent antioxidant responses in the lung [142]. Similar studies revealed that sputum concentrations of GSH and GSSG are increased in healthy smokers [143] and also in patients with stable (moderate to severe) COPD [144]. However, ELF levels of GSH are decreased in COPD patients with frequent exacerbations [145], suggesting that oxidative stress in these cases result from pro-inflammatory states during exacerbations rather than from chronic smoking. Moreover, lung tissue or ELF concentrations of persulfidated forms of GSH or cysteine (e.g., glutathione persulfide, GSSH; cysteine persulfide, CysSSH; and glutathione trisulfide, GSSSH), which represent oxidized forms of GSH or cysteine due to persulfidation reactions involving H_2_S [146], are also decreased in COPD patients in correlation with airflow limitation [147]. The authors speculated that this may be due to impaired synthesis of H_2_S even though expression of some enzymes involved in H_2_S biosynthesis was actually enhanced.

Similar to the discussion above related to the FRT of ageing, a major limitation with respect to the concept of oxidative stress or redox imbalance in COPD is the dynamic nature of ROS production (be it from CS or from endogenous sources), as well as their diverse biological actions, which makes overall assessment of their contributions to ageing or age-related diseases such as COPD extremely complicated. In this regard, the potential role(s) of NADPH oxidases in either ageing or in COPD is relatively unexplored, and this forms the main focus of the next sections.

## 6. NADPH Oxidases (NOX) in Lung Physiology and Pathology

The NADPH oxidase (NOX) enzyme family is widely distributed throughout metazoans, and generates ROS (O_2_^−^ or H_2_O_2_, depending on the NOX homolog) in a tightly regulated and deliberate fashion to participate in a wide range of biological processes, including chemical host defense, regulation of cell proliferation and differentiation, immune regulation, and hormone synthesis. The enzymology and biology of NOX enzymes has been extensively reviewed elsewhere [148,149,150], and will be only briefly summarized here. The NOX family comprises seven genes in mammals (NOX1-5 and DUOX1/2, although NOX5 is lost in rodents) which are widely distributed throughout the organism [151,152]. First recognized for their role in phagocytic cells in oxidative host defense, as part of the so-called respiratory bursts, NOX enzymes are now broadly recognized as ‘professional’ ROS generators for purposes of redox-based cell signalling, largely through reversible protein oxidation on e.g., cysteines [153,154,155], or for extracellular hormone synthesis or matrix remodelling via peroxidase enzymes as partner proteins [156]. All NOX enzymes contain a C-terminal intracellular dehydrogenase domain (with NADPH and FAD-binding sites) and six transmembrane segments anchoring two heme groups, that mediate transmembrane electron transfer from NADPH/FADH_2_ to molecular O_2_. NOX1-4 all require an additional transmembrane protein (p22^phox^) and (in case of NOX1-3) various cytosolic co-factors that need to assemble with the transmembrane protein complex for enzyme function. NOX5 does not require additional cofactors but contains an intracellular calmodulin-like domain with four calcium binding EF-hand structures. The dual oxidases DUOX1 and 2 also contain intracellular EF-hand calcium binding domains, as well as an additional extracellular protein domain with homology to heme peroxidases which lack peroxidase function, but are required for optimal maturation and targeting to the cell surface.

NOX enzymes are widely distributed throughout the lung and expressed in diverse cell types within the lung (Table 2). Some NOX homologs are expressed constitutively and may be involved primarily in maintenance of cellular homeostasis. Other NOX isoforms (e.g., DUOX2, NOX4) are more readily inducible during e.g., infection or in response to e.g., growth factors and may play greater roles in host defense, or tissue development or remodelling. However, this distinction is not absolute, and all NOX enzymes have important homeostatic functions, whereas inappropriate induction or activation of NOX is also likely to contribute to the pathology of various (age-related) diseases. In this regard, NOX enzymes have been considered as an excellent example of the antagonistic pleiotropy hypothesis [82].

The most well-known NOX homologue, NOX2, is primarily present in phagocytic cells, and critical for their antimicrobial function [161]. However, NOX2 is also important in regulating inflammation [162] and NOX2 deficiency can lead to unregulated inflammation, thus resulting in chronic granulomatous disease (GCD) [163]. Given its role in ROS production during phagocyte activation, NOX2 is thought to contribute to the pathology of chronic lung diseases associated with inflammation, such as COPD, but studies with NOX2-deficient mice have given mixed results (see also below). NOX2 is furthermore present in structural lung cells, including endothelial and epithelial cells and fibroblasts, and contributes to epithelial or endothelial activation during bacterial or viral infection [164,165,166]. NOX1 has been shown to be involved in regulation of airway barrier function [167], and contributes to apoptosis during hyperoxia and has been shown to be involved in acute lung injury [168]. NOX4 is involved in various cellular functions including oxygen sensing, cell proliferation and differentiation, apoptosis, fibrosis, and inflammation [169,170,171]. An excessive expression of NOX4 was reported in pulmonary diseases, including pulmonary fibrosis, pulmonary hypertension, and COPD [172,173,174].

The DUOX enzymes are primarily expressed in bronchial and alveolar epithelial cells within the lung, and produce H_2_O_2_ in response to various stimuli to serve mucosal anti-microbial and anti-viral host defense functions [175,176]. Indeed, DUOX1 was recently demonstrated to possess antiviral properties against influenza in mice, which was related to H_2_O_2_-dependent production of hypothiocyanite (OSCN^−^) by lactoperoxidase in airway epithelial secretions [177]. Studies from our group implicated a role for DUOX1 in epithelial responses to non-infectious injurious triggers, and identified a contribution of DUOX1 in epithelial wound healing by redox signaling. During such injurious triggers, DUOX1 is activated by calcium-dependent signaling due to initial damage signals such as ATP that activate purinoceptor (P2YR2) signalling, as well as voltage-gated Ca^2+^ channels or transient receptor potential (TRP) channels [178]. In turn, DUOX1 activation mediates redox-dependent activation of tyrosine kinases such as Src or EGFR [176,179,180], pathways that are involved in the induction of wound genes such as matrix metalloproteinase (MMP)-9 or the neutrophil chemokine IL-8, but also in rapid epithelial secretion of alarmin cytokines (interleukins IL-1α and IL-33) that are critical in inducing appropriate immune responses [181]. These initial responses activate type 2 immune processes that involve recruitment and activation of type 2 innate lymphoid cells (ILC2s) and other Th2 cells [182], that play critical roles in epithelial host defenses by promoting barrier function, restoration of epithelial integrity and homeostasis, and mucus production [176]. Indeed, chronic allergic airway diseases such as asthma are characterized by upregulation of airway DUOX1 as a contributing factor to heightened Th2 responses to allergen challenge, and increased mucus hyperplasia and airway remodelling as major features of asthma pathology [176]. In contrast to the apparent association of DUOX1 with host responses to non-microbial injury, microbial infections appear to be associated with dramatic induction of airway DUOX2 rather than DUOX1 [183,184], and DUOX2 has been implicated in airway antibacterial or antiviral host defense associated with TLR activation and activation of Th1 immune responses [176].

The diverse and specific functional properties of different NOX enzymes in the lung would also predict that they may also be variably involved in lung dysfunction associated with ageing or in COPD pathology, and these questions are only beginning to be addressed.

## 7. NOX Enzymes and Their Relation to Normal Ageing

While ageing is known to be associated with redox alterations and oxidative stress (perhaps largely related to mitochondrial dysfunction), much less is known with respect to the associations of ageing and NOX enzyme expression or function. Some associations between NOXes and ageing have been summarized in recent reviews [11,185], but many knowledge gaps remain. Among the earliest reports addressing the effect of age on NOX expression are findings of age-related increases in NOX4 expression in vascular smooth muscle cells [186,187]. Analysis of GTEx database data also indicated age-related increases in NOX4 in human lung tissues [65]. Curiously, another study found that whole lung NOX4 mRNA levels were downregulated with age [188]. Although NOX4 did not appear to affect longevity in mice [188], age-related increases in NOX4 have been associated with alterations in genes involved in TGF-beta (TGF-β) signalling and extracellular matrix remodelling [65], consistent with a role for NOX4 in age-related pulmonary fibrosis [189,190]. Age-related increases NOX4 are also thought to be responsible for elevated ROS production and increased endothelial cell permeability in the context of acute lung injury [191].

From the perspective of age-related changes in immune cell biology, a number of studies have addressed the impact of age on NOX2, the main NOX enzyme in immune cells. Indeed, an age-related loss of NOX2 was observed in human peripheral mononuclear cells and was found responsible for age-related dysfunction of immunosuppressive T cells (CD8+CCR7+ Tregs), thereby potentially promoting pro-inflammatory responses and increasing susceptibility to chronic inflammatory diseases [192]. A related study demonstrated that NOX2 deficiency, through impaired development of Th17/Treg cells, spontaneously induced arthritis development in mice, of which the severity proportionally increased with age [193]. Endothelial senescence induced by the matricellular protein thrombospondin 1 (TSP1), an important feature of vascular ageing, was found to be associated with induction and activation of NOX1, and could be attenuated by targeted NOX1 inhibition [194,195].

Our group recently surveyed alterations of lung tissue NOX mRNA levels in mice of advancing age. While we did not observe significant changes with respect to NOX1, NOX2, or NOX4, we noted a marked decline in lung DUOX1 mRNA and protein with advancing age. Analysis of human lung tissues in the GTEx dataset indicated a significant negative association of lung DUOX1 transcripts with age. Accordingly, we also observed that DUOX1-mediated innate airway epithelial injury responses to external non-microbial triggers, such as house dust mite allergen, were dramatically impaired with age [29]. Age-related DUOX1 downregulation would also be expected to lead to impaired DUOX1-mediated antiviral responses [177], which may be relevant for the increased susceptibility of ageing individuals to viral infections [196,197] such as influenza, and possibly also SARS-CoV-2 [198].

In spite of these various observations of altered NOX expression during aging, it is often unclear whether these alterations also contribute to specific features of ageing. Intriguingly, a role for DUOX1 in longevity was inferred from studies of the nematode *C. elegans*, which contain a NOX isoform Ce-Duox1 (also known as blistered-3; BLI-3), which shares 30% of its amino acid sequence with human DUOX1 [199]. Studies using RNA interference (RNAi) deletion indicated that BLI-3 can promote longevity, due to its ability to generate ROS and activate the Nrf2 homolog SKN-1 to enhance oxidative stress resistance [200,201,202]. Moreover, *C. elegans* that carry a dysfunctional BLI-3 mutation (either in the BLI-3 peroxidase domain or NADPH oxidase domain) are short-lived [203]. Whether DUOX1 deficiency also affects longevity in mammals is unknown, but our recent studies suggest that DUOX1 deficiency in mice can accelerate some features of lung aging, illustrated by enhanced age-related senile emphysema and associated lung function changes [29]. However, DUOX1 deficiency did not significantly affect other common hallmarks of aging, such as senescence markers or SASP, or ageing-related airway or alveolar matrix remodelling. It is also unclear whether any of the other NOX enzymes affect mammalian lifespan. No such role was observed for NOX4 [188], and although NOX1 appears to contribute to endothelial senescence [194], its impact on overall lifespan is not known. However, an intriguing recent report indicated that deletion of NADPH oxidase organizer 1 (NOXO1), a cofactor for NOX1, resulted in longer lifespan in mice, which was postulated to be related to improved DNA repair capacity in NoxO1-deficient mice [204].

In aggregate, NOX enzymes appear to play some role(s) in various hallmarks of ageing in the lung, such as genomic (in)stability, (altered) intercellular communication and cellular (and immuno)senescence. However, the fact that ageing may have opposing effects on different NOX enzymes further illustrates the difficulty in connecting redox alterations with specific hallmarks of ageing. Finally, it also is worth mentioning that ageing is associated with changes in the NAD(P)H status [205], which is not only relevant for redox homeostasis mediated by NADPH-dependent oxidoreductases, but also for appropriate NOX function as it requires NADPH. Alterations in NAD(P)H status have also been associated with mitochondrial dysfunction and increased mtROS production [206,207].

## 8. NOX Enzymes in COPD Pathology

Similar to the relative lack of available studies addressing associations of NOX enzymes with ageing, a rather limited number of previous studies have attempted to address the specific role(s) of NOX enzymes in COPD pathology. Not surprisingly, increased numbers of NOX2-positive inflammatory cells have been observed in lung tissues from COPD patients, and a contributing role of macrophage NOX2 in elastase-induced emphysema has been reported using NOX2-deficient mice [208]. However, while NOX2 contributes to macrophage-mediated oxidative stress and inflammation due to cigarette smoke exposure [209], genetic deletion of NOX2 in mice was actually found to aggravate CS-induced emphysema, which was associated with increased inflammation that was perhaps worsened due to NOX2 deficiency [162,210]. These discrepant findings may be related to the different animal models used, variable roles of NOX2 in limiting chronic inflammation through e.g., Nrf2 [162] or promoting injury during acute inflammation (e.g., in the case of the elastase model), and diverse functions of NOX2 in different cell types, which would be best dissected using cell- or tissue-specific NOX2 knockout strategies. Some studies have reported elevated levels of NOX4 in airway smooth muscle of COPD patients [211], which were found to correlate with disease severity [212] and to be associated with pulmonary hypertension [213]. Furthermore, RTP801/REDD1, which negatively regulates mammalian target of rapamycin (mTOR), is upregulated in response to cigarette smoke, and enhances inflammation and alveolar destruction by increasing NOX4 activity [214]. In a genetic mouse model of emphysema (due to TLR4 deficiency), elastolytic activity was found to be increased due to induction of NOX3 in the pulmonary endothelium and resultant oxidant generation [215], but the relevance for human COPD is unclear.

**Figure 1:**
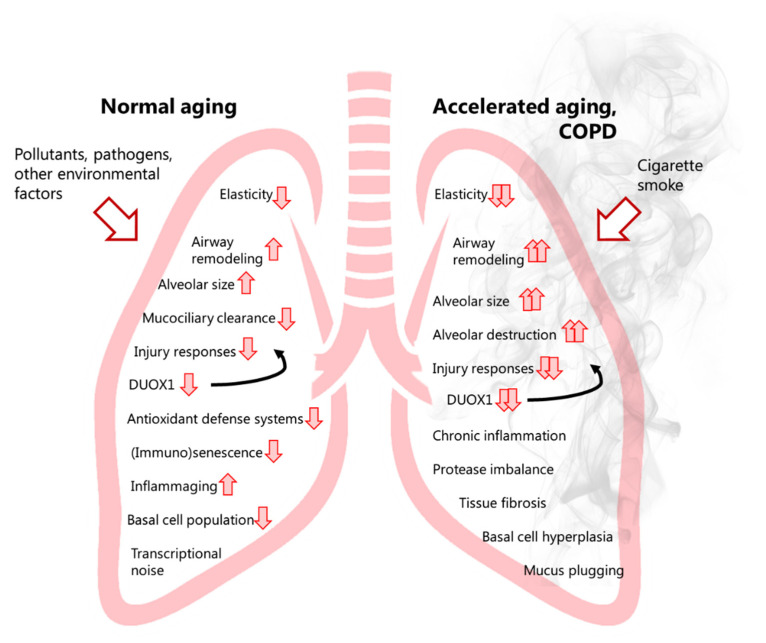
Normal lung ageing versus accelerated lung aging in COPD, with a role for the NADPH oxidase dual oxidase 1 (DUOX1). Various recognized events of normal lung aging (left lung) are described, with additional events occurring only during accelerated lung ageing such as COPD (right lung). The recognized impact of ageing and COPD on DUOX1 and its influence on airway remodeling, lung elasticity, alveolar enlargement, mucociliary clearance and regenerative responses are illustrated therein.

Analysis of tracheal and bronchial epithelium collected by airway brushing or laser capture micro-dissection, revealed that DUOX1 was significantly suppressed in the airways of healthy smokers and patients with COPD, when compared to age-matched control subjects, implying that chronic smoking leads to decreased airway epithelial expression of DUOX1 as a potential contributing factor in COPD development [216,217]. Our group expanded on these findings, by showing a gradual downregulation of DUOX1 protein in the small airways of GOLD II-IV COPD patients, which was found to be strongly correlated with various measures of lung function decline, and several markers of small airway remodeling and destruction [218]. On one hand, these results may imply that variable DUOX1 downregulation as a result of normal aging (see above) or smoking history may predispose for COPD development and progression. Alternatively, it is also possible that DUOX1 downregulation may be a consequence of COPD, for example secondary to production of inflammatory mediators or growth factors such as TGF-β. Indeed, we observed that chronic exposure of bronchial epithelial cells to TGF-β also downregulates DUOX1 [218]. Downregulation of DUOX1 may be related to smoking history, although some studies suggest that exposure to CS extract can actually enhance DUOX1 expression [219]. In contrast, chronic exposure of mice to the CS-component acrolein was found to result in DUOX1 downregulation, a response that could be mimicked by chronic in vitro exposure of epithelial cells to acrolein [218]. Nevertheless, the relationship between airway or alveolar DUOX1 and smoking status/history is complex, and observed correlations of airway DUOX1 with lung function parameters in our recent studies were largely independent of smoking status [218], suggesting the contribution of other factors to DUOX1 downregulation in COPD. To address a potential causal effect of DUOX1 down-regulation in COPD development or progression, we assessed the impact of DUOX1 deletion in a mouse model of elastase-induced emphysema or in a mouse model of small airway remodeling due to chronic acrolein exposure. In both cases, we observed worse disease phenotypes in DUOX1-deficient mice suggesting that DUOX1 down-regulation in COPD may actively contribute to disease progression, likely related to altered epithelial biology and homeostasis, as well as neutrophilic inflammation and degranulation [218]. Moreover, in light of recent work indicating a role for DUOX1 in antiviral innate immunity [177], decreased DUOX1 in the lung of COPD patients may also promote susceptibility to viral infection and may thereby enhance exacerbations [220]. Overall, the apparent roles of NOX enzymes in COPD pathology are variable, with some NOX enzymes (e.g., NOX2 and NOX4) contributing to aspects of COPD pathogenesis, whereas others (most notably DUOX1, Figure 1) may actually help to prevent COPD progression. This has important implications for the proposed contribution of oxidative stress to COPD, and the use of antioxidant strategies to treat COPD or other ageing-related diseases, which will be further discussed more specifically in the next section.

## 9. Therapeutic Targeting of Oxidative Stress in COPD: Current Status and Pitfalls

Based on the prevailing notion that redox imbalance or oxidative stress contribute to features of ageing and age-related chronic lung diseases such as COPD, several therapeutic approaches have been proposed to target oxidative stress or redox imbalance as potential treatment for COPD (Table 3) [221,222]. Given the aforementioned sections highlighting the high diversity in ROS sources (either environmental or endogenous, including various NOX enzymes that are regulated independently) and their specific associations with distinct redox-based protein modifications and signaling events, it should not be surprising that approaches to globally manipulate redox homeostasis or inhibit oxidative events have been only minimally effective in managing age-related diseases such as COPD.

Therapeutic approaches to target oxidative stress can roughly be divided into 2 categories. First, small molecule antioxidant compounds or antioxidant micronutrient supplements are used in an attempt to directly quench ROS and prevent their ability to oxidize critical cell constituents. However, in addition to concerns related to biological distribution and bioavailability, such approaches may not distinguish between harmful and beneficial actions of ROS. Moreover, it is also important to emphasize that, even though these compounds are typically lumped together as “anti-oxidants”, their chemical mechanisms of action and ability to react with different ROS species are highly variable. A second group of “anti-oxidant”-based therapeutics work by promoting endogenous anti-oxidant responses rather than by direct anti-oxidant mechanisms. These include compounds that enhance endogenous redox enzyme status (e.g., small molecule Nrf2 activators such as sulforaphane) or compounds that enhance cellular levels of redox co-factors NAD(P) or NAD(P)H (e.g., NAD^+^ precursors) to support redox homeostasis by optimizing the function of NADPH-dependent oxidoreductases. A third group of compounds are molecules that directly target specific cellular sources of ROS (e.g., mitochondria, NOX enzymes), which would theoretically allow for distinguishing harmful effects of some specific ROS sources from beneficial effects associated with e.g., NOX-dependent redox signaling. Indeed, the recent discovery of diverse NOX enzymes with diverse functional properties has fueled a search for isoform-specific NOX inhibitors, some of which are now in clinical development [223]. A separate category of compounds that does not function as an antioxidant per se but may work to prevent or reverse (oxidative) damage induced by senescent cells, are known as senolytics or senostatics, the latter working to block accumulation of senescent cells by inhibiting paracrine cell signaling [224]. The unfortunate habit of lazily grouping these various compounds together under the rubric “anti-oxidants” has done a disservice to the field of redox biology, as it does not acknowledge the specificity of actions of different ROS species by diverse (enzymatic) sources. Oftentimes compounds are defined as “anti-oxidant” even though they have highly pleiotropic biochemical effects, and it is highly uncertain whether their cellular effects are due to their purported anti-oxidant properties. The flavonoid quercetin is a good example of this, as it has been claimed it to be an anti-oxidant [225], whereas others also refer to it as a senolytic [226], and yet others define it as a Src kinase inhibitor [227]. Although some of these activities may be mechanistically related, it would be premature to attribute all biological actions of quercetin to its putative anti-oxidant properties. In other cases the term anti-oxidant is completely inappropriate, for example in case of Nrf2 activators which are often electrophilic compounds that work by modifying thiol groups, which reflects a pro-oxidant rather than a direct anti-oxidant mechanism. In the next paragraphs, we will summarize the current status with respect to the clinical use of some of these anti-oxidant-based approaches in COPD, and will also discuss the various limitations and pitfalls in light of the recent developments with respect to NOX biology in the context of aging or age-related disease.

### 9.1. Supplementary “Antioxidants”

Among the first anti-oxidant-based approaches for COPD is the thiol-compound N-acetyl-cysteine (NAC), which was originally developed as a mucolytic agent that reduced mucus viscosity. As a thiol species, NAC is also thought to act as a direct anti-oxidant or as a precursor of cysteine to boosting GSH synthesis. However, the “anti-oxidant” actions of NAC in experimental studies are rarely established, and recent studies suggest that NAC may also serve as a precursor of H_2_S and sulfane sulfur species [228]. Nevertheless, NAC and similar thiol-based compounds such as erdosteine, carbocysteine, and fudosteine, have been extensively examined in COPD, and various clinical studies have indicated some efficacy [222,229]. Indeed, some clinical trials have shown successes of NAC treatment in the outcome of various parameters (e.g., reduced acute exacerbations, mortality risk, hospitalization), but others have shown NAC treatment to be largely ineffective (no change in FEV_1_, lung function) [92,230]. Other thiol-based compounds (e.g., carbocysteine) have been shown to decrease COPD exacerbations in clinical trials, although they did not improve lung function [231,232]. Based on this, some of these thiol-based compounds are now used clinically for the treatment of COPD in Europe and Asia [222]. However, it is important to emphasize that the beneficial effects of these compounds is most likely related to their mucolytic properties rather than by their suggested ‘anti-oxidant’ mechanisms [233].

Another class of compounds in this category are anti-oxidant mimetics, such as metalloporphyrins and manganese-containing molecules that are used as SOD mimetics, or glutathione peroxidase mimetics such as ebselen. Although no studies have reported their clinical efficacy in COPD patients, some of these mimetics have performed well in phase I safety studies and are currently being developed for COPD patients [234].

Since it is well-recognized that poor diet presents an important risk factor for many chronic diseases including COPD [235,236], and diet is an importance source of anti-oxidant vitamins such as vitamins C (ascorbic acid) or E (α-tocopherol), carotenoids or flavonoids, it has been speculated that enhancing dietary anti-oxidant intake might benefit COPD patients. However, although some studies have associated vitamin anti-oxidant status with indices of lung function, vitamin supplementation studies have so far not shown to significantly improve lung function or clinical features of COPD [237]. A more recent randomized placebo-controlled trial of nutritional anti-oxidant supplementation (α-tocopherol, ascorbate, zinc gluconate, selenomethionine) in COPD patients during pulmonary rehabilitation did not reveal improved muscle endurance, although it did significantly improve of muscle strength and other training outcomes [238].

Although anti-oxidant supplementation, especially dietary anti-oxidants, is often deemed harmless, it is important to point out some limitations and caveats associated with such supplementation strategies. In addition to issues related to bioavailability and distribution, preclinical and clinical studies have reported significant adverse health effects which may be especially relevant for subject groups with existing smoking related co-morbidities. Indeed, among the most famous early examples is a large randomized trial of a cohort of Finnish male smokers which received nutritional anti-oxidant supplements (beta-carotene and alpha-tocopherol) which demonstrated increased incidence of lung cancer specifically in subjects receiving beta-carotene [239]. Subsequent preclinical studies more directly highlighted similar adverse effects of anti-oxidant supplementation. For example, NAC supplementation in a mouse model of COPD was found to decrease lung oxidative damage, cellular senescence, and emphysema, but increased development of lung adenocarcinoma [240]. Similar studies in genetic mouse models of lung cancer showed that anti-oxidant supplementation (e.g., NAC, or vitamin E) can enhance lung cancer progression and metastasis [241]. Also, while recent studies indicate the potential of Trx as a modulator of COPD pathology [242], Trx1 overexpression also has the potential to enhance tumor development [77]. Taking into consideration that COPD patients are often at increased risk for the development of lung carcinoma [243], it would seem prudent to exercise caution when considering anti-oxidant-based supplementation strategies in management of COPD.

### 9.2. Small Molecule Activators of Endogenous Anti-Oxidant Responses

In addition to directly supplementing anti-oxidants, other small molecules can promote redox homeostasis or anti-oxidant defense more indirectly, such as activators of the endogenous anti-oxidant response mediated by Nrf2. Examples of such Nrf2 activators are sulforaphane and CDDO-Im, and recent evidence indicates that such approaches can mitigate inflammation, improve innate antibacterial defenses, and restore corticosteroid-induced responses in COPD patients [244,245]. Specifically, sulforaphane treatment rescued phagocytosis and bacterial recognition by alveolar macrophages isolated from COPD patients.

Furthermore, nicotinamide adenine dinucleotide (NAD^+^) is a hydrogen carrier for redox enzymes with important roles in redox homeostasis and has emerged as a critical signaling molecule and essential substrate for sirtuins (SIRT), a class of enzymes that mediate several beneficial effects related to ageing [246], including inhibition of senescence and telomere attrition, sustaining genome integrity and improved DNA damage repair. Interestingly, NAD^+^ levels are known to be decreased during ageing in multiple model organisms [247], and a loss of sirtuin-1 and sirtuin-6 are observed in COPD [248,249]. Additionally, since NADPH is a critical co-factor for various reducing enzymes as well as redox homeostasis, the loss of NAD^+^ levels during ageing may also negatively impact on such NADPH-dependent processes. For example, NAD^+^ is thought to fuel NADPH for various oxidoreductases including glutathione reductase or thioredoxin reductase. There is now emerging in vivo evidence suggesting that restoring NAD^+^ levels in old or diseased animals may promote health and extend lifespan [250]. Additionally, a recent systematic review identified that NAD supplementation may be useful in age-related conditions [251]. Therefore, NAD^+^ restoration through e.g., NAD precursors may be a potential therapy during ageing and in COPD.

While some of these therapeutics are already in use or may be potential new strategies in age-related lung pathologies, it is difficult to predict how such treatment strategies including nutritional agents, Nrf2 activators, and NAD precursors, may be applicable to the ageing population in absence of lung pathology. Intriguingly, the ageing hallmarks are specifically established as features of ageing that are present in absence of pathology, and age-related lung pathologies seem to follow the concept that there is an acceleration of these processes. Thus, there may be value in preventing (Nrf2 activators) or retarding (senolytics, supplemental antioxidants) the acceleration of various hallmarks that induce age-related lung pathology, thereby promoting ‘healthy’ ageing of the lung, which may potentially increase quality of life and lifespan [252].

### 9.3. Inhibitors of ROS Production

The antioxidant-based approaches described above are designed to directly detoxify ROS or improve overall redox homeostasis but are unable to distinguish between different biological actions of ROS from distinct cellular sources. Indeed, because mitochondria are a major cellular sources of ROS, and mitochondria are dysfunctional in COPD [123], mitochondria-targeted anti-oxidant compounds have been developed (mitoQ, mitoTEMPO) and are in clinical development for various age-related diseases (e.g., [234]). Some preclinical studies have indeed suggested beneficial effects of mitoQ against COPD pathology [125], but no clinical studies of mitoQ in COPD have yet been reported. Relevant to the central theme of this review, pharmacological inhibitors of NOX enzymes have been used in several preclinical studies, for example apocynin, which has been shown to prevent chronic CS-induced skeletal muscle mass loss and function in mice [253]. However, clinical application of commonly used pharmacological NOX inhibitors has been limited because of lack of specificity and concerns about toxicity. In light of the discovery of multiple NOX enzymes with unique functional properties, there has been major effort to develop NOX-selective inhibitors, some of which (e.g., setanaxib) are currently in clinical development ([223]). Importantly, although some NOX-selective inhibitors may be beneficial in the context of specific lung pathologies [176,254], recent observations of reduced function of e.g., DUOX1 in the context of ageing or COPD may also limit the application of NOX inhibitors in COPD and would instead argue for therapeutic approaches that enhance DUOX1-specific function.

## 10. Conclusions, and Future Perspectives

The original concept that oxidative stress is a major contributor to normal ageing and age-related disease has not resulted in significant clinical progress with respect to antioxidant-based strategies. Indeed, several biologically important functions of ROS in host defense and health span has refined our view on this topic [47], and it is now increasingly appreciated that non-discriminating approaches to target ROS would also inadvertently impact on their beneficial effects, e.g., redox signaling events, especially those that are linked with the widely conserved family of NOX enzymes. This may also help explain why systematic reviews of anti-oxidant supplementation studies have failed to demonstrate substantial effects of anti-oxidant supplementation with respect to preventing chronic disease, and in fact even suggest that anti-oxidant supplementation can even enhance mortality [255], which is perhaps due to unwanted interference with such beneficial redox processes. Recent observations that functionality of some specific NOX enzymes may actually be impaired with ageing or during age-related diseases such as lung cancer or COPD [218] would call for further caution and may actually warrant alternative strategies aimed at enhancing specific ROS-mediated processes, rather than generic ROS-inhibiting approaches. Indeed, the general attitude of “there is no harm in trying” with respect to anti-oxidant supplementation may be reasonable for young and healthy individuals in preventing disease, but may be less recommendable for subjects with underlying health conditions, especially those with increased cancer risk.

A recent review that summarizes the various targeted therapeutic approaches for COPD, including several anti-oxidant approaches [242], perfectly illustrates the underlying problem: many diverse pathways are being considered for targeting, and there is likely no single therapeutic strategy that will effectively mitigate COPD. In this context, the generic ROS-targeting strategies discussed above are also unlikely to be sufficiently effective. One major caveat in the search for suitable drug targets for COPD is the relative absence of faithful animal models, which are often based on studies in young rodents and do not recapitulate potentially important contributions of normal ageing. Additionally, differences in metabolic rates and disease progression of such animal models further complicate the optimal timing at which such therapeutic intervention should be initiated. In fact, many studies with animal models tend to test potential therapeutics during the developmental stage of the experimentally induced disease rather than in a more relevant therapeutic fashion, which further limits the translatability of such findings.

As mentioned earlier, the recent observations of impaired expression or function of some NOX enzymes during ageing or COPD would not support the use of anti-oxidant-based strategies or NOX inhibitors, but would instead suggest that experimental approaches are needed to enhance the expression or function of these NOX enzymes. In this regard, it is tempting to speculate that current approaches of NAD precursors, that are currently being promoted to extend healthy ageing, may also work by enhancing cellular NADPH status [205], and thereby promote the functional activity of NOX (e.g., [256]). Moreover, pharmacological approaches that would help sustain or enhance NADPH pools, e.g., by enhancing the activity of NAD kinase [205,257] or glucose 6-phosphate dehydrogenase (G6PD) [257], may be beneficial in promoting health span and could also help prevent age-related disease, in part by promoting NOX function, although approaches targeting specific NOX enzymes would be preferred due to the potential opposing effects of promoting overall NOX function. Interestingly, these enzymes are also being explored as therapeutic targets in treatment of cancer [258,259], which further illustrates the dichotomy of redox-modifying agents having potentially beneficial effects with respect to prolonging ageing whereas they may also promote cancer progression. In this regard, personalized medicine approaches guided by patient-specific alterations in expression of NOX or other enzymes involved in redox homeostasis may form the best strategy to inform appropriate therapeutic targeting approaches for subjects with COPD.

## 11. Patents

AvdV is coinventor on U.S. Patent No. 10143718, “Covalent Inhibitors of Dual Oxidase 1 (DUOX1),” issued 4 December 2018.

## Figures and Tables

**Table 1:** Table summarizing the experimental evidence for and against the free radical theory of aging.

The Free Radical Theory (FRT) of Aging [8]	
Supporting evidence	Conflicting evidence
Strong correlations between chronological age and levels of reactive oxygen species (ROS) generation and oxidative stress markers [48,49,50,51]	Lack of correlation between levels of ROS and longevity across various species [52,53,54]
Age-related accumulation of irreversible oxidation products in proteins or DNA [55,56,57,58,59] Increased DNA damage and lipid peroxidation products in aged vs. young organisms [58,59]	Mutant organisms associated with mitochondrial dysfunction have prolonged lifespan [60,61] Mitochondrial hormesis: relatively low levels of mitochondria-derived ROS improve systemic defense mechanisms and promote healthspan [62,63]
Age-related aggregation of mitochondrial damage may cause accumulated mutations in mitochondrial DNA [64] Biological ageing is associated with decreased expression of antioxidant and oxidoreductase enzymes [65] Gender divergence in mice during ageing associated with oxidative stress [66] Ageing is associated with decreased adaptive response to oxidative stress [67] Age-dependent diseases are frequently associated with increased oxidative stress [68]	Studies with antioxidant supplementation strategies typically do not enhance longevity and can sometimes reduce it [69] Genetic manipulation of antioxidant defense genes in mice does not affect lifespan [70,71] Some oxidative protein cysteine modifications may be important for promoting longevity and decrease with ageing [10] Hydrogen sulphide (H_2_S) may positively impact lifespan and ageing through S-sulfhydation (or persulfidation) [72]

**Table 2:** Expression profile of NADPH oxidase homologs in the lung.

	Normalized Expression	Lung Cell Expression [157]	ROS Production
NOX1	0.2	Endothelium Immune cells	O_2_^•−^
NOX2/CYBB	49.4	Endothelium Immune cells Alveolar and airway epithelium	O_2_^•−^
NOX3/gp91phox	0.1	Endothelium N.A.	O_2_^•−^
NOX4	1.2	Smooth muscle and endothelium Myofibroblasts Immune cells Alveolar and airway epithelium	O_2_^•−^ H_2_O_2_
NOX5	0.6	N.A.	O_2_^•−^
DUOX1	48.7	Alveolar and airway epithelium	H_2_O_2_
DUOX2	1.2	Alveolar and airway epithelium	H_2_O_2_

Normalized expression (NX); based on data from three sources: Human Protein Atlas (HPA) RNA-seq data, RNA-seq data from the Genotype-Tissue Expression (GTEx) project and CAGE data from FANTOM5 project in the lung according to the RNA Consensus dataset [158,159,160], with respective lung cell expression profiles and ROS production. A cut-off value of 1 NX was used as a limit for detection across all tissues or cell types. ROS: reactive oxygen species; N.A.: not available.

**Table 3:** Various approaches targeting oxidative stress in COPD that are currently tested for use, and potential novel strategies.

Compounds	Examples	Pitfalls	Findings
Thiol compounds with mucolytic properties	N-acetyl-cysteine (NAC) Erdosteine Carbocysteine Fudosteine	Most likely function as mucolytics, rather than ROS scavengers	Clinical studies indicated some efficacy (reduced exacerbations, mortality risk, hospitalization); others did not (no improvement in lung function) [92,222,229,230,231,232]
Mimetics of glutathione peroxidase superoxide dismutase	Ebselen (Manganese) Metaloporphyrins	These ‘anti-oxidants’ do not scavenge all ROS	Clinical efficacy in COPD patients unknown; Performed well in phase I safety studies; Currently being developed for COPD patients [234]
Dietary agents, polyphenols	Ascorbate α-tocopherol Carotenoids Flavonoids Zinc gluconate Selenomethionine	Not necessarily effective against all ROS; Some flavonoids (e.g., quercetin) may function as senolytics, potentially by tyrosine kinase inhibition; Flavonoid oxidation to (semi) quinones may generate electrophiles that activate Nrf2	Mixed findings; No improvement in lung function, clinical features of COPD [237]; Improved muscle strength [238]; Adverse health effects: increased incidence of lung cancer [239]
Nrf2 activators	CDDO-Im Sulforaphane	Do not directly target ROS	Alleviates inflammation; improves innate antibacterial defenses; restores corticosteroid-induced responses [244,245]
Potential Strategies	Examples	Potential Pitfalls	Findings
Mitochondria-targeted anti-oxidant compounds	MitoQ MitoTEMPO	May interfere with mtROS signaling	No clinical knowledge in COPD yet
Activators of endogenous anti-oxidant responses	Nicotinamide adenine dinucleotide (NAD) precursors	May also enhance NOX function	No clinical knowledge in COPD yet
Pharmacological inhibitors of of NADPH oxidase enzymes	Apocynin Setanaxib	Inhibition of DUOX1 function	No clinical knowledge in COPD yet; Setanaxib currently in clinical trial for IPF [223]

## Data Availability

Data is contained within the article.

## Abbreviations

The following abbreviations are used in this manuscript:

AP	Antagonistic pleiotropy
ATP	Adenosine triphosphate
BLI	Blistered cuticle
CGD	Chronic granulomatous disease
COPD	Chronic obstructive pulmonary disease
CS	Cigarette smoke
CT	Computerized tomography
CysSSH	Cysteine persulfide
DAMP	Danger-associated molecular pattern
DNA	Deoxyribonucleic acid
DUOX	Dual oxidase
ECM	Extracellular matrix
EcSOD	Extracellular superoxide dismutase
EELV	End-expiratory lung volume
EGF	Epidermal growth factor
EGFR	Epidermal growth factor receptor
ELF	Epithelial lining fluid
EMT	Epithelial mesenchymal transition
FAD/FADH2	Flavin adenine dinucleotide
FEV1	Forced expiratory volume in 1 s
FRC	Functional residual capacity
FRT	Free radical theory
FVC	Forced vital capacity
GOLD	Global initiative for chronic obstructive lung disease
GSH	Glutathione
GSSG	Glutathione disulfide
GSSH	Glutathione persulfide
GSSSH	Glutathione trisulfide
GTEx	Genotype-tissue Expression
G6PD	Glucose-6-phosphate dehydrogenase
H_2_O_2_	Hydrogen peroxide
H_2_S	Hydrogen sulfide
IL	Interleukin
ILC	Innate lymphoid cell
IPF	Idiopathic pulmonary fibrosis
JNK	C-jun N-terminal kinase
Mclk1	Mitochondrial 5-demethoxyubiquinone hydroxylase
MDA	Malondialdehyde
MMP	Matrix metalloproteinase
MnSOD	Manganese superoxide dismutase
MPO	Myeloperoxidase
mtDNA	Mitochondrial deoxyribonucleic acid
mTOR	Mechanistic target of rapamycin
NAC	N-acetyl cysteine
NADPH	Nicotinamide adenine dinucleotide phosphate
NADH/NAD^+^	Nicotinamide adenine dinucleotide
NF-κB	Nuclear factor kappa B
NOX	NADPH oxidase
NOXO1	NADPH oxidase organizer 1
Nrf2	Nuclear factor erythroid 2–related factor 2
Nuo-6	NADH dehydrogenase ubiquinone flavoprotein 1
O_2_^•−^	Superoxide
OH^•^	Hydroxyl radical
OSCN^−^	Hypothiocyanite
RBC	Red blood cell
ROS	Reactive oxygen species
SASP	Senescence-associated secretory phenotype
SERPIN	Serine protease inhibitor
SOD	Superoxide dismutase
SIRT	Sirtuin
TGFβ	Transforming growth factor β
Th1	T-helper type 1
Th2	T-helper type 2
Th17	T-helper type 17
TLR	Toll-like receptor
Treg	Regulatory T-cell
Trx	Thioredoxin
TSP1	Thrombospondin 1
